# Treatment of tungiasis with a two-component dimeticone: a comparison between moistening the whole foot and directly targeting the embedded sand fleas

**DOI:** 10.1186/s41182-017-0046-9

**Published:** 2017-03-10

**Authors:** Per Nordin, Marlene Thielecke, Nicholas Ngomi, George Mukone Mudanga, Ingela Krantz, Hermann Feldmeier

**Affiliations:** 1 The Skaraborg Institute for Research and Development, Stationsgatan 12, 541 30 Skövde, Sweden; 2 Institute of Microbiology and Hygiene, Campus Benjamin Franklin, Charité University Medicine, Berlin, Germany; 3 African Population and Health Research Center, Nairobi, Kenya; 4 Department of National Disease Control, Ministry of Health, Kampala, Uganda; 5 Epidemiology and Global Health, Department of Public Health and Clinical Medicine, Umeå University, Umeå, Sweden

**Keywords:** Tungiasis, Treatment, Dimeticone, Public health

## Abstract

**Background:**

Tungiasis (sand flea disease) is caused by the penetration of female sand fleas (*Tunga penetrans*, Siphonaptera) into the skin. It belongs to the neglected tropical diseases and is prevalent in South America, the Caribbean and sub-Saharan Africa. Tungiasis predominantly affects marginalized populations and resource-poor communities in both urban and rural areas. In the endemic areas, patients do not have access to an effective and safe treatment. A proof-of-principle study in rural Kenya has shown that the application of a two-component dimeticone (NYDA®) which is a mixture of two low viscosity silicone oils caused almost 80% of the embedded sand fleas to lose their viability within 7 days.

**Methods:**

In this study we compared the efficacy of two distinct modes of application of NYDA®; one targeted application to the area where the parasite protrudes through the skin and one comprehensive application to the whole foot.

**Results:**

Independent of the two modes of application, the dimeticone caused more than 95% of embedded sand fleas to lose all signs of viability within 7 days. The targeted application killed embedded sand fleas more rapidly compared to when the whole foot was covered. The proportion of viable lesions at day two were 7.0 versus 23.4% (*p* < 0.01) and at day five 3.9 versus 12.5% (*p* < 0.02).

**Conclusions:**

Our findings suggest that the dimeticone could provide a safe and effective treatment for tungiasis in areas with difficult access to health care.

**Trial registration:**

ISRCTN ISRCTN74306878

## Background

Tungiasis (sand flea disease) belongs to the family of neglected tropical diseases and is prevalent in South America, the Caribbean and sub-Saharan Africa [1]. It is caused by penetration of female sand fleas (*Tunga penetrans*) into the skin and the ensuing inflammatory response [2]. The inflammation is intensified by an almost unavoidable bacterial super-infection [3]. Just about all of the lesions are found in the feet [4, 5]. The consequences of sand flea disease are debilitating, eventually leading to chronic morbidity with impaired mobility and quality of life [6, 7].

The prevalence of tungiasis varies between settings; prevalences up to 60% have been reported in various populations with up to 80% in children [8–11]. Children and the elderly are more likely to develop severe disease [4, 11, 12]. Tungiasis predominantly affects marginalized populations and people living in resource-poor communities in both urban and rural areas [8, 10, 13–15].

Various drugs have been examined for their efficacy against embedded sand fleas in humans. Randomized controlled trials using topical or oral administration of antihelminthic drugs such as metrifonate, thiabendazole or ivermectin showed little or no efficacy at all [16–19].

The only treatment option patients in the endemic areas have is to try to kill embedded sand fleas chemically or mechanically. People often apply toxic substances such as kerosene, used engine oil or household insecticides. Alternatively, the lesions are manipulated with sharp instruments such as needles, safety pins, razor blades or thorns, a health risk by itself. Such practices can also cause additional bacterial super-infections or transmit viral pathogens such as HBV, HCV and HIV [7].

The last three abdominal segments of an embedded sand flea form a miniature cone through which the parasite remains in contact with the environment through an opening in the skin of about 250 μm. Through this opening, the female sand flea takes up oxygen, expels eggs, defecates and gets fertilized. The abdominal cone protrudes through the skin and has been identified as a target for drug treatment [20, 21]. As the skin around the abdominal cone is painful, patients usually know exactly how to localize an embedded sand flea.

A proof-of-principle study in rural Kenya has shown that the application of a two-component dimeticone (NYDA®) to the skin of the feet, repeated two times within 5 min, kills almost 80% of the embedded fleas within 7 days [21]. Furthermore, assessments of lesion morphology indicate that normal development was interrupted in those parasites not killed: the female fleas became unable to produce and/or expel eggs. Lesion-associated inflammation significantly decreased within 7 days after application of dimeticone [21].

NYDA® contains two dimeticones or silicone oils with different viscosities and a high creeping property. It is commercialized as a medical device for the treatment of head lice infestation in many European countries [22, 23]. Its mode of action is purely physical [24].

In this study, we compared the efficacy of two distinct modes of application of NYDA®: one targeted application to the area where the abdominal cone of the parasite protrudes through the skin and one general application to the whole foot.

The rationale for the targeted application was twofold: first, to minimize the volume of the dimeticone and, second, to direct the dimeticone to where it should act, namely the vital organs of the parasite located inside the abdominal cone. By consequence, a targeted application of the dimeticone should lead to a more rapid death of an embedded sand flea.

## Methods

The study took place from the end of February till the end of March 2014, i.e. during the end of the dry season, when transmission of tungiasis peaks.

### Study area

The study was conducted in eight primary schools in Bugiri district, Bulidha sub-county, eastern Uganda. The schools were located in the following villages: Makoma, Isaka Bisolo, Kibuye, Businda, Busakira, Nakawa and Wakawaka.

### Study population

Sixty children aged 5 to 12 years selected from eight primary schools were included in the study. The number of children sampled from each school was based on the school and class size as well as on the organizational convenience. The number of children enrolled per school varied from 3 to 21.

Children from classes one to six present at the day of the investigation were eligible for the study provided they had at least three viable sand flea lesions on each foot (stage 2 and/or 3 according to the Fortaleza classification [25]) as evaluated by a rapid assessment method [26].

They should furthermore not show clinical symptoms requiring immediate medical attention such as abscesses, ulcers or intense pain. When multiple embedded sand fleas of stage 2 or 3 were present, only lesions which could be clearly distinguished from one another were included in the study.

**Fig. 1:**
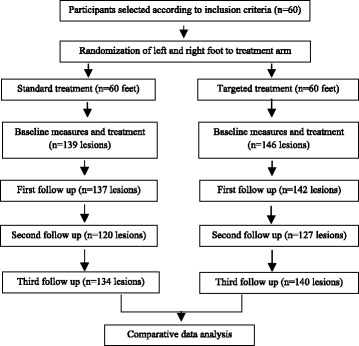
Sampling of the patients, allocation of feet to treatment arms and number of lesions included

Lesions were chosen so that their location made it possible to use the handheld digital microscope, such as at the toe, the sole or the rim of the foot. Each lesion was photographed, and its location and stage was noted in the patient’s record. The number of lesions included in the study was limited to three per foot and were followed up at regular intervals for 7 days (Fig. 1).

### Study design

Two topical regimens of the two-component dimeticone (NYDA®) (Pohl-Boskamp GmbH & Co. KG, Hohenlockstedt, Germany) were randomly allocated to either the left or the right foot of the patient. Before the application of dimeticone, both feet were washed with water and soap and dried with a clean towel.

The regimens were labeled ‘whole foot treatment’ and ‘targeted treatment’. Whole foot treatment meant that dimeticone was applied to the skin of the foot up to the ankle, as previously described [21]. The application was stopped when the skin became shiny, indicating that it was wetted with the dimeticone. This procedure was repeated three times within 10 min and required 2 to 5 ml depending on the size of the foot.

Targeted treatment meant that dimeticone was aspired into a 5-ml syringe to which a flexible tube was mounted. Three drops were applied to the area where the parasite’s abdominal cone protruded through the skin. One drop corresponds to approximately 50 μl of dimeticone. This procedure was repeated three times within 10 min to ensure that a maximum amount of dimeticone entered into the abdominal cone of the parasite within a short period of time. This required approximately 450 μl per embedded sand flea. In both groups, the dimeticone was only applied at baseline.

For each patient, demographic data as well as baseline parasitological measurements were conducted as previously described [21, 25, 27]. Staging was performed according to the Fortaleza Classification.

- Stage I: penetrating sand flea
- Stage II: brownish-black dot with a diameter of 1-2 mm
- Stage III: circular yellow-white watch glass-like patch with a diameter of 3–10 mm and with a central black dot
- Stage IV: brownish-black crust with or without surrounding necrosis

Stage I to III are viable sand fleas; in stage IV, the parasite is dying or already dead [25].

Using a digital handheld microscope (dnt DigiMicro Mobile 5-megapixel-handheld-microscope, ITEZ, Hong Kong, China) viability signs (expulsion of eggs, excretion of faeces threads, excretion of liquid, pulsations/contractions) were recorded as present or not.

### Outcome measures

All data were collected by the same investigator at baseline and during a 7-day follow-up: day two, day five and day seven.

Two major outcome measures were defined: the viability of the embedded sand flea and the intensity of the local inflammation. The primary outcome measure was the viability of the embedded sand flea according to the Fortaleza Classification [25]. An embedded sand flea was considered to be dead, if none of the four viability signs was detected during 15 min of observation by the digital handheld microscope on two consecutive follow-up examinations [20]. A secondary outcome measure was the intensity of the local inflammation, as assessed semi-quantitatively by an inflammation score [21]. Lesions manipulated by the patient or the caregiver were also documented.

Another set of outcome measures were based on visual scales depicting how the impact of tungiasis was perceived by the patient. The scales consist of a series of simple pictures illustrating itching, pain, itching-related sleep disturbance, pain-related sleep disturbance and mobility impairment as perceived by the patient. The patient was asked to classify the degree of each complaint by pointing to the corresponding picture. Zero meant no complaint at all, 1 = little complaint, 2 = moderate complaint, 3 = severe complaint and 4 = very severe complaint. These outcome measures encompass both feet, since it was considered impossible for the participants to discern the impact of embedded sand fleas separately for each foot.

### Statistical methods

The sample size of circa 140 lesions per treatment group is based on a 15% difference in treatment effect, a power of 90% and a significance level of 5%. The assumption was based on our previous findings [21]. Fisher’s exact test was used to compare proportions, and the Kruskal-Wallis test was used to analyze the inflammation score and self-reported tungiasis-related characteristics. A relationship was considered statistically significant when a *p* value was less than 5%. All presented confidence interval (CI) have a confidence level set at 95%.

## Results

### Baseline

At baseline, the two treatment groups displayed similar characteristics concerning the distribution of lesion types (Table 1).

**Table 1:** Types of lesions at baseline in the two treatment groups

	Whole foot treatment		Targeted treatment	
Lesion type^a^	Median	(Min–max)	Median	(Min–max)
Viable lesion	4	(1–75)	5	(1–50)
Non-viable lesion	4	(0–30)	4	(0–30)
Manipulated lesion	1	(0–12)	1	(0–15)
All lesions	9	(1–94)	10	(2–70)

^a^According to definition in subjects and methods

The difference between the number of lesions found on the two feet for the same individual were never larger than 12 in all cases except one. In this particular case, the foot intended for whole foot treatment had 75 lesions and the foot intended for targeted treatment only nine lesions. The distribution of differences in number of lesions between the paired feet, i.e. feet on the same individual are considered a pair, showed a mean of 0.1 together with a standard deviation of 9.7, and the related median had a value of 0. The average numbers of viable lesions were close to seven per foot with a substantial variation in both treatment groups. The two groups also displayed a similar distribution of frequencies across the range of the parasites’ developmental stages (Table 2).

**Table 2:** Number of viable lesions per developmental stage of lesions in the two treatment groups at baseline

Stage^a^	Whole foot treatment	Targeted treatment
2a	30	39
2b	96	93
3a	12	12
3b	1	2
Total number	139	146

^a^According to the Fortaleza classification [25]

### During and after treatment

**Fig. 2:**
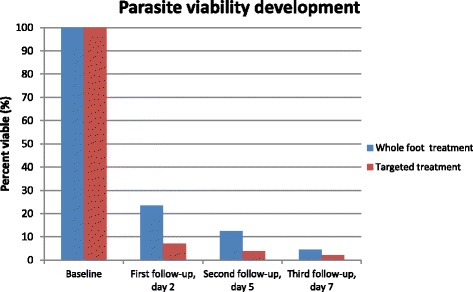
Decline of parasite viability from baseline with treatment for each group through the three follow-up examinations at day 2, day 5 and day 7

The outcome measured as the loss of viability of embedded sand fleas after the application of the dimeticone is shown in Fig. 2.

At the first follow-up, 2 days after treatment, the number of viable parasites decreased significantly in both groups (whole foot treatment *p* < 0.001; targeted treatment *p* < 0.001). The loss of viability was higher in the targeted treatment group (*p* < 0.001). At the second follow-up, after 5 days, the loss of viability was still higher in the targeted treatment group compared to the whole foot treatment group (*p* < 0.02). After 7 days, the reduction of the number of viable sand fleas was similar in both groups: 95% (CI 92; 99) of the parasites in the whole foot treatment group had lost all signs of viability and 97% (CI 94; 99) in the targeted treatment group (Table 3). Furthermore, in both groups, sand fleas which remained viable did not expel eggs during the 7 days. Whether lesions were in stage 2 or 3 at baseline had no impact on the efficacy of either treatment (Table 3).

**Table 3:** Viability of embedded sand fleas in the treatment groups at baseline and the subsequent follow-ups^a^

		Whole foot treatment			Targeted Treatment			
	Lesion stage^b^	No. viable	No. non-viable	Viable (%)	No. viable	No. non-viable	Viable (%)	*p* value
Baseline (day 0)	All lesions	139	0	100	146	0	100	n.a.
	stage 2	126	0	100	132	0	100	n.a.
	stage 3	13	0	100	14	0	100	n.a.
First follow-up (day 2)	All lesions	32	105	23.4	10	132	7.0	<0.001
	stage 2	31	92	25.2	8	116	6.5	<0.001
	stage 3	1	13	7.1	2	16	11.1	0.600
Second follow-up (day 5)	All lesions	15	105	12.5	5	122	3.9	0.018
	stage 2	14	95	12.8	5	109	4.4	0.030
	stage 3	1	10	9.1	0	13	0	0.458
Third follow-up (day 7)	All lesions	6	128	4.5	3	137	2.1	0.326
	stage 2	5	115	4.2	3	120	2.4	0.496
	stage 3	1	13	7.1	0	17	0.0	0.452

*n.a*. not applicable

^a^Not all participants and all lesions could be examined at every occasion, which explains differences in the numbers of examined lesions during follow-ups

^b^According to the Fortaleza classification [25]

The inflammation scores and the visual scale measurements showed significant reductions between baseline and day seven (Table 4).

**Table 4:** Secondary outcome measures at baseline and at day seven for both feet combined

	Baseline						Day 7				
Outcome	N	Median	IQR^a^	Min	Max	N	Median	IQR^a^	Min	Max	*p* value^b^
Inflammation score	56	4.3	3.4	1	22	57	0.5	1.5	0	12	<0.001
Visual scales											
Intensity of spontaneous pain	60	3	1	1	4	60	0	1	0	4	<0.001
Intensity of itching	60	3	1	1	4	60	0	1	0	3	<0.001
Itch-related sleep disturbance	60	2	1	1	4	60	0	1	0	4	<0.001
Pain-related sleep disturbance	60	2	1	1	4	60	0	0	0	3	<0.001
Degree of mobility impairment	52	2	1	1	4	59	0	1	0	4	<0.001

^a^Interquartile range

^b^Kruskall-Wallis test

After completion of the study (day 7), the medians of all visual scales had decreased to 0.

## Discussion

Tungiasis is endemic in resource-poor populations in many countries of sub-Saharan Africa (8, 10, 13, 14, 15). It is associated with important morbidity, and children and the elderly carry the highest disease burden (4, 11, 12). Nonetheless, hitherto there is no approved treatment. In Kenya, the Ministry of Health recommends bathing the feet for 10 min in KMnO4, an approach with a rather low efficacy [21]. Besides, KMnO4 stains the skin in deep purple. This makes the treatment visible for everyone and the patient vulnerable to ridiculosity [21]. Hence, there is an urgent need for a safe and effective treatment of tungiasis.

The study showed that with the targeted application of the dimeticone, parasites were killed more rapidly and that 2 days after the topical application, only 7% of the parasites remained viable (Table 3). After 7 days, though, in both treatment groups, >95% of the embedded sand fleas had lost all viability signs. A more rapid death of the parasites is an advantage, because inflammation resolves as soon as an embedded sand flea has died (Thielecke M, unpublished observation 2014, [28]).

It is likely that by a repeated targeted application, more dimeticone crept into the abdominal cone per unit of time compared to when the skin of the whole foot is wetted and that this resulted in a rapid death.

Actually, when looking at the abdominal cone with the digital microscope, one could see how the dimeticone creeps into the opening in the skin and then disappears, as it spreads to the microscopic surfaces located within the abdominal cone. A similar observation has been made when dimeticone is applied to free running insects such as head lice and crickets [23].

Seven days after the application of the dimeticone, 98% of the parasites had lost all their viability signs in the targeted treatment group and 95.5% in the whole foot treatment group. Only a few sand fleas withstand the treatment, but these were not observed to expel eggs during the remainder of the study, indicating that the dimeticone had abrogated the normal development of the female sand flea as observed previously [21]. The implication of this finding is that a scaled-up treatment program could have an effect on the transmission.

Tungiasis-related symptoms decreased rapidly, when the sand fleas lost their viability by the dimeticone. This was visible both as indicated by the inflammation score assessed by the investigator, as well as by the visual scales as expressed by the patients themselves (Table 4). Notably, the median became 0 7 days after treatment in all visual scales. One week after treatment, healing was evident as seen by the decrease in the inflammation score as well as the visual scales confirming a previous finding [21].

During a previous study, we have observed that the inflammation around the embedded sand fleas increase when they continue to grow and intensify their metabolic activity in stages 2 and 3 [27]. The inflammation recedes, however, when the parasites develop from stage 3 to 4 [25]. In these stages, the signs of viability slowly disappear and eventually the parasites die.

The efficacy of the whole foot treatment here was higher compared to the proof-of-principle study in Kenya where dimeticone applied to the feet up to ankles killed 78% of the embedded sand fleas within 7 days [21]. Apart from the difficulty of achieving a standardized application procedure giving a precise dosage for each foot, there are also biological explanations for this difference. Rapid penetration of the dimeticone into the last abdominal segments of the embedded sand flea may depend on how deep a parasite is located in the skin. The thicker the corneal layer of the skin, the longer the dimeticone will need to reach the stratum of the epidermis in which the parasites are located. In addition, only a part of the dimeticone will be absorbed by a rough and thickened epidermis. The children in Kenya did not use shoes at all, whereas the children in Uganda, at least partially, had sandals or flip-flops. Thus, the higher efficacy of the dimeticone in this study might reflect a thinner and smoother corneal layer of the feet of the Ugandan participants.

Lesions localized to the tip of the toes, the sole and the rim of the foot were deliberately chosen so that the handheld digital microscope easily could be applied in order to assess viability signs with a higher degree of precision. At those selected sites, it can be suspected that the dimeticone might be targeted more precisely and/or penetrate the parasite more rapidly. No such selections were made in the Kenyan study. There, the included lesions were also located under the nail, under thick crusts of the corneal layer or in necrotic tissue areas into which the dimeticone cannot penetrate easily. A difference in penetration efficiency could thus possibly also explain the differing results.

The number of lesions varied considerably within the group of included children (Table 2). When the number of lesions are more than three, they often occur in clusters [29] making it difficult to distinguish the characteristics of an individual lesion, which also impairs assessments of morphological changes and the lesion-associated inflammation [21]. We, therefore, deliberately limited the number of included lesions to three per foot.

The results of this study are based on highly controlled and monitored procedures. This means one ought to be cautious when considering the true effectiveness of treatment of tungiasis with dimeticone under field conditions. Based on the average required volume of dimeticone for treatment of the whole foot and what is used in the targeted treatment, we find that the targeted application is more parsimonious as long as the number of viable embedded sand fleas does not exceed seven lesions per foot. Hence, in severe cases where individuals suffer from dozens of embedded viable sand fleas, the whole foot treatment should be more cost-effective and also more practicable.

## Conclusions

The application of a mixture of a two-component dimeticone (NYDA®) caused more than 95% of embedded sand fleas to lose all defined signs of viability within 7 days. The targeted topical application worked faster compared to when the whole foot was covered. Our findings suggest that the dimeticone could provide a safe and effective treatment for tungiasis in areas with difficult access to health care.

## Abbreviations

CI: Confidence interval
HBV: Hepatitis B virus
HCV: Hepatitis C virus
HIV: Human immunodeficiency virus
ISRCTN: International standard randomised controlled trials number
WHO: World Health Organization
